# American Medical Association

**Published:** 1866-06

**Authors:** 


*American Medical Association.*—The following officers were reported at the recent meeting of this body held in Baltimore: President, H. F. Asken, of Delaware; Vice-Presidents, W. K. Bowling, of Tennessee; J. C. Hughes, of Iowa; H. J. Bowdich, of Massachusetts; Thos. C. Brinsmore, of New York; Permanent Secretary, Wm. B. Atkinson, of Pennsylvania; Assistant Secretary, W. W. Dawson, of Cincinnati; Treasurer, Casper Wistar, of Pennsylvania. Cincinnati is the place for next annual meeting.

